# Demonstration of stimulated emission depletion phenomenon in luminescence of solid-state scintillator excited by soft X-rays

**DOI:** 10.1038/s41598-020-62100-0

**Published:** 2020-03-25

**Authors:** Takeo Ejima, Toshitaka Wakayama, Natsumi Shinozaki, Misaki Shoji, Genta Hatayama, Takeshi Higashiguchi

**Affiliations:** 1 Institute of Multidisciplinary Research for Advanced Materials, Tohoku University, 2-1-1 Katahira, Aoba-Ku, Sendai 980-8577 Japan; 2 School of Clinical Engineering, Saitama Medical University, 1397-1 Yamane, Hidaka, Saitama 350-1241 Japan; 3 Department of Electrical and Electronic Engineering, Faculty of Engineering, Utsunomiya University, 7-1-2 Yoto, Utsunomiya, Tochigi 321-8585 Japan

**Keywords:** Imaging techniques, Optical sensors, Imaging techniques, Imaging techniques, Imaging and sensing

## Abstract

Although imaging techniques using soft X-rays (SXs) are being developed as the available photon flux increases because of the continuing development of synchrotron light sources, it will be necessary to downsize the pixel size of the SX camera to produce finer SX images. Application of the stimulated emission depletion (STED) method to a scintillator plate followed by use of this plate as a sensor is one promising method to reduce the pixel size of SX cameras. A STED phenomenon occurred in the luminescence of a Ce-doped Lu_2_SiO_5_ crystal (Ce:LSO) excited using ultraviolet (UV) light when the scintillator was irradiated with azimuthally polarized laser light in the photon energy range from 1.97 eV (630 nm) to 2.58 eV (480 nm). When the excitation light source changed to synchrotron radiation (SR) light with photon energy of 800 eV, the same STED phenomenon occurred. The spot size of the luminescence was reduced by the STED phenomenon and this spot size decreased as the STED laser’s photon energy increased. The energy dependence of the Ce:LSO luminescence levels can be used to explain the change in the spot size at the luminescence point.

## Introduction

One of the criteria used for evaluation of digital cameras is the image sensor used in the device. In general, a larger image sensor size allows a wider field of view to be acquired, while a smaller pixel size enables acquisition of finer images. The wide variety of image sensors that are available also leads to a wide variety of cameras, from the cameras installed in handy smartphones to high-end single-lens reflex (SLR) cameras. The situation is the same for X-ray image acquisition. In the soft X-ray (SX) region with its energy range from 30 eV to 3 keV, back-illuminated charge-coupled device (CCD) sensors are used as the image sensors. At present, commercially available CCD cameras for the SX region have pixel dimensions of 13.5 × 13.5 μm^2^ and contain 1024 × 1024 pixels. These values have remained constant for the past 10 years and we have no alternative options for image sensor configuration^1^. Because of this lack of options, the freedom of X-ray optical systems equivalent to that of camera lenses has narrowed; therefore, the methods available for X-ray measurement have been limited to date. As the types of available X-ray image sensors increase, the latitude of X-ray optical systems will increase accordingly and a variety of X-ray measurement methods will then be obtained.

Scintillators show luminescence in the visible wavelength region when exposed to high-energy light, e.g., X-rays or γ-rays, and these devices are widely used as two-dimensional (2D) detectors in applications such as computed tomography equipment^2^. In a 2D detector, the scintillator plate converts an X-ray image into a visible (VI) image, which can then be magnified using a VI microscope. In recent years, some scintillators have demonstrated the stimulated emission depletion (STED) phenomenon under UV light excitation, such as Tb:LSO scintillators, and the possibility of realising a super-resolving microscope using a scintillator has been proposed^3^. If this STED phenomenon can be applied to detection of the luminescence image of the scintillator plate, the spatial resolution of the luminescence image will increase and exceed the Abbe diffraction limit^4^.

**Figure 1:**
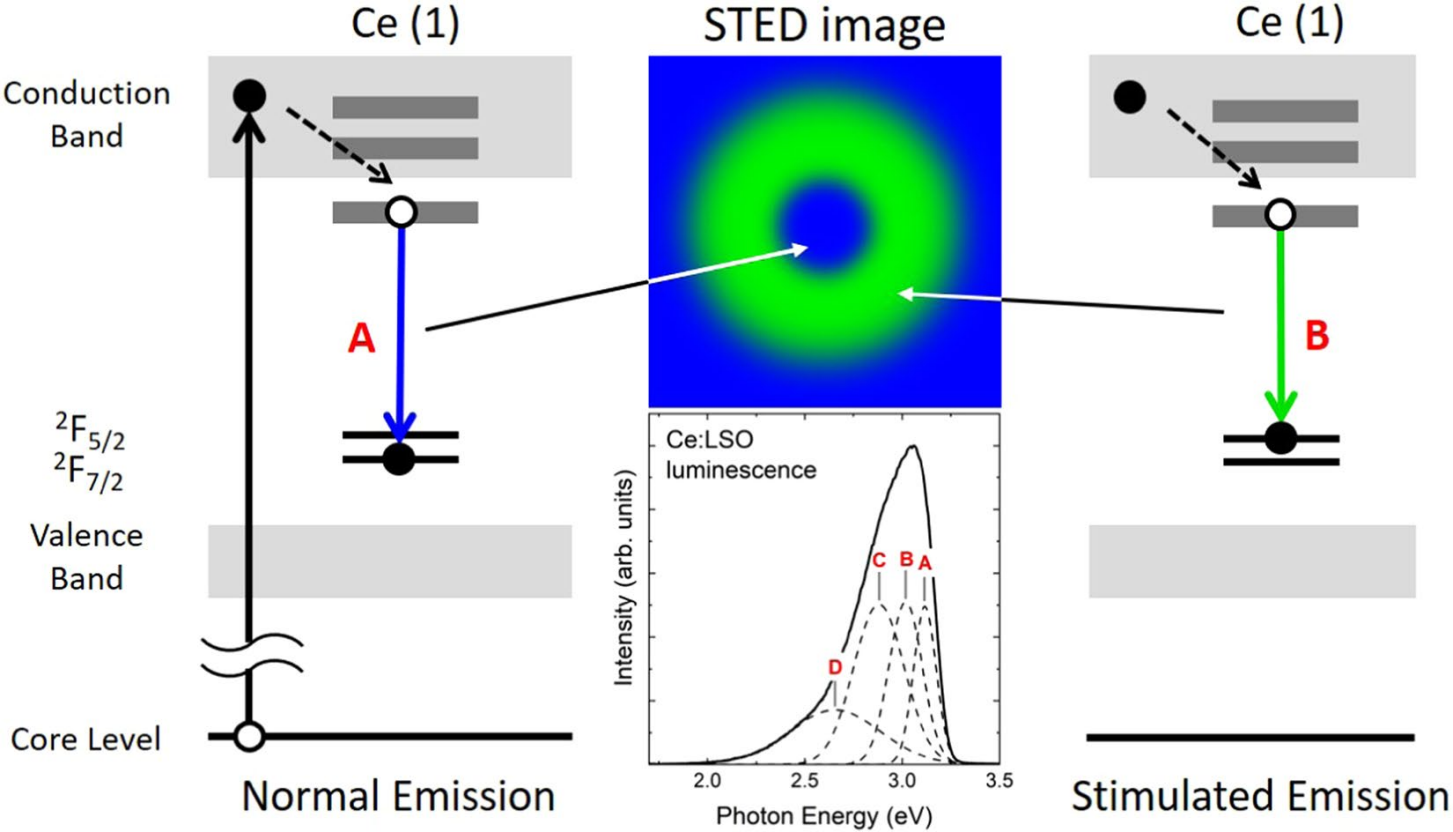
Possibility of STED phenomenon in Ce:LSO when excited by SX light. In the LSO crystal, two Lu atom sites exist and are denoted by Lu(1) and Lu(2)^7^. The Ce atoms that replace the Lu atoms at the Lu(1) and Lu(2) sites are also denoted by Ce(1) and Ce(2), respectively, in the figure.

One scintillator, which is composed of a Lu_2_SiO_5_ crystal doped with cerium atoms (Ce:LSO), is a phosphor that exhibits luminescence over a wide energy range from the soft X-ray (SX) to hard X-ray (HX) regions^5^. The Ce:LSO luminescence spectrum shows its maximum luminescence intensity at a photon energy of approximately 3.10 eV (*λ* = 400 nm) and shows a tail structure on the long-wavelength side from the peak^2^. Because the Lu atoms in the LSO crystal are located at two different sites and the Ce atoms that are introduced randomly displace Lu atoms, the Ce atoms are thus also coordinated to the two different sites^1^. The Ce^3+^ ions have two occupied Ce 4 *f* states: the ^2^*F*_5/2_ and ^2^*F*_7/2_ orbitals, as shown in Fig. 1 ^6^. The dominant transitions of the luminescence are from the lowest unoccupied Ce 5*d* state to the occupied Ce 4 *f* states. Therefore, the Ce:LSO luminescence spectrum shows four different luminescence peaks: the two peaks that originate from the site difference and the other two peaks that originate from the ionization levels of the Ce ions. In this paper, we demonstrate the STED phenomenon in the luminescence of Ce:LSO that has been excited by SX light and the area limits of the luminescence region using vector light at the weak luminescent wavelength of Ce:LSO.

The spatial resolution of a STED microscope is represented by the modified Abbe diffraction limit^4^. When the spatial resolution *δd*_*N*_ of an ordinary objective lens is given by the Abbe diffraction limit, *δd*_*N*_ = *λ*/2NA, where NA is the numerical aperture of the objective lens and the observation wavelength is *λ*, the spatial resolution *δd*_STED_ given by the modified Abbe diffraction limit is

1 $$\frac{\delta {d}_{{\rm{STED}}}}{\delta {d}_{N}}=\frac{1}{\sqrt{1+{I}_{{\rm{STED}}}/{I}_{{\rm{sat}}}}},$$

where *I*_sat_ is the fluorescent saturation intensity of the phosphor and *I*_STED_ is the light intensity of the stimulated emission light at the focal plane. Hereinafter, ${I}_{{\rm{STED}}}/{I}_{{\rm{sat}}}$ will be referred to as the normalized laser intensity. The best reported spatial resolution for the STED microscope to date is approximately 6 nm, recorded when observing nitrogen impurities in diamond^4^.

## Results

### Ce:LSO luminescence spectrum

The luminescence spectra of Ce:LSO, when excited using 800 eV SX light, were measured at room temperature (RT) and at the liquid nitrogen temperature (LNT). The spectral shape obtained was similar to the previously reported spectral shapes^5^. When the luminescence spectrum shape obtained at RT was compared with that of the spectrum obtained at the LNT, the two spectra showed almost the same profile, apart from the shoulder structure at the LNT in the figure, which was designated peak B. A difference between the sample temperatures caused the shoulder structure to disappear.

**Figure 2:**
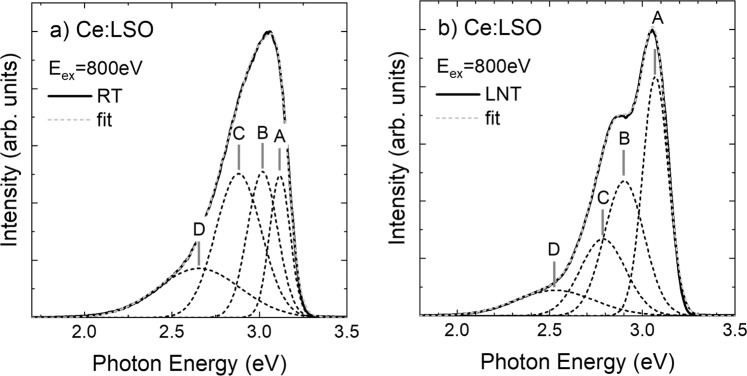
Luminescence spectrum of Ce:LSO obtained by SX excitation at (**a**) LNT and (**b**) RT. Solid curves show the measurement results and broken curves represent the fitting results.

Next, the spectra obtained were deconvoluted using Gaussian peaks. As described in the Introduction, the peak structure was expected to comprise four luminescence levels; therefore, the luminescence spectra were deconvoluted and separated according to expectations. The deconvoluted curves are distinguished by broken curves in Fig. 2 and Table 1 summarises the parameters of the peaks.

**Table 1:** Peak profiles of the deconvolution results in the luminescence spectra of Ce:LSO shown in Fig. 2.

Quantity	A	B	C	D
Energy @RT (eV)	3.1	3.0	2.9	2.7
FWHM @RT (eV)	0.14 ± 0.01	0.20 ± 0.03	0.31 ± 0.03	0.55 ± 0.02
Integrated Int. @RT	16.3	24.5	36.6	22.6
Peak Int. @RT	0.50 ± 0.02	0.51 ± 0.07	0.50 ± 0.06	0.17 ± 0.02
Energy @LNT (eV)	3.1	2.9	2.8	2.5
FWHM @LNT (eV)	0.16 ± 0.01	0.25 ± 0.1	0.3 ± 0.3	0.50 ± 0.06
Integrated Int. @LNT	36.1	31.6	20.6	11.8
Peak Int. @LNT	0.84 ± 0.02	0.47 ± 0.5	0.27 ± 0.6	0.09 ± 0.06

### Stimulated emission depletion by UV excitation

Over the range from 2.58 eV (480 nm) to 1.97 eV (630 nm), measurements were taken to verify whether or not the luminescence of the Ce:LSO was depleted and to determine the excitation photon energy of the STED phenomenon under SX excitation conditions. The occurrence of the depletion phenomenon was confirmed over the photon energy region from 2.58 eV to 2.25 eV.

**Figure 3:**
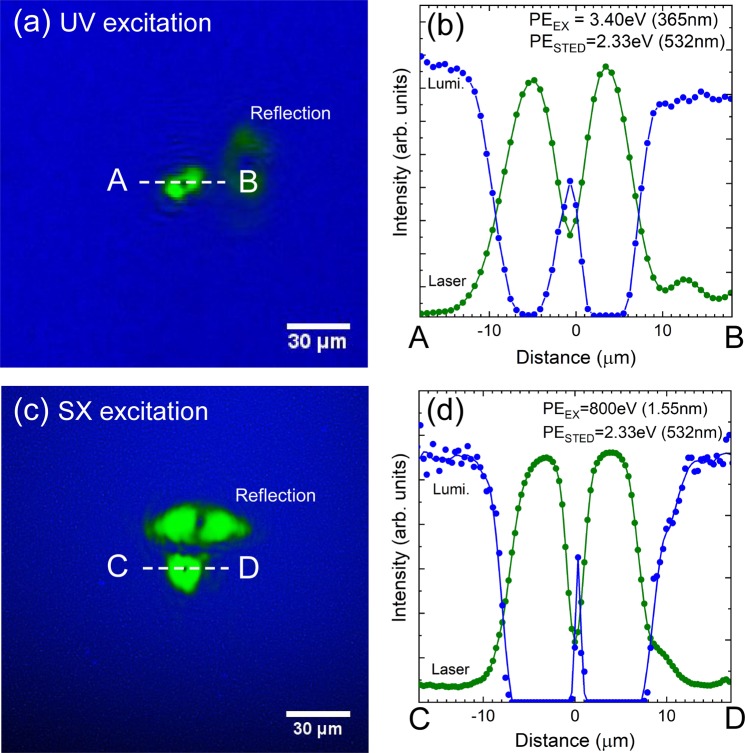
Luminescence image of UV excitation with the spot of the azimuthally polarized laser light. (**a**) Beam profiles at the broken line A–B. (**b**) Luminescence image of SX excitation (**c**), and the beam profiles at the broken line C–D. (**d**) In all figures, green colour represents the laser spot, and blue colour is the luminescence of the scintillator. In (**a**,**c**), “Reflection” means the reflection beam spot of the STED laser from the scintillator surface.

Because the STED light drawn as the green area in the figure was azimuthally-polarized vector light, there is no central light intensity in this spot. The blue area represents the luminescence from the Ce:LSO that was excited by the UV light. The excitation light illuminated the entire scintillator plane and thus the entire observed area emitted blue light, apart from the area illuminated using the STED laser light. Figure 3(b) shows the light intensity profiles of the luminescence and the STED light beams indicated by the line A-B in Fig. 3(a). The maximum and minimum values are normalized with respect to the profile curves. The luminescence intensity varies in accordance with the increase and reduction of the STED light intensity and the excitation light and luminescence light intensity profiles have shapes that are the inverse of each other.

**Figure 4:**
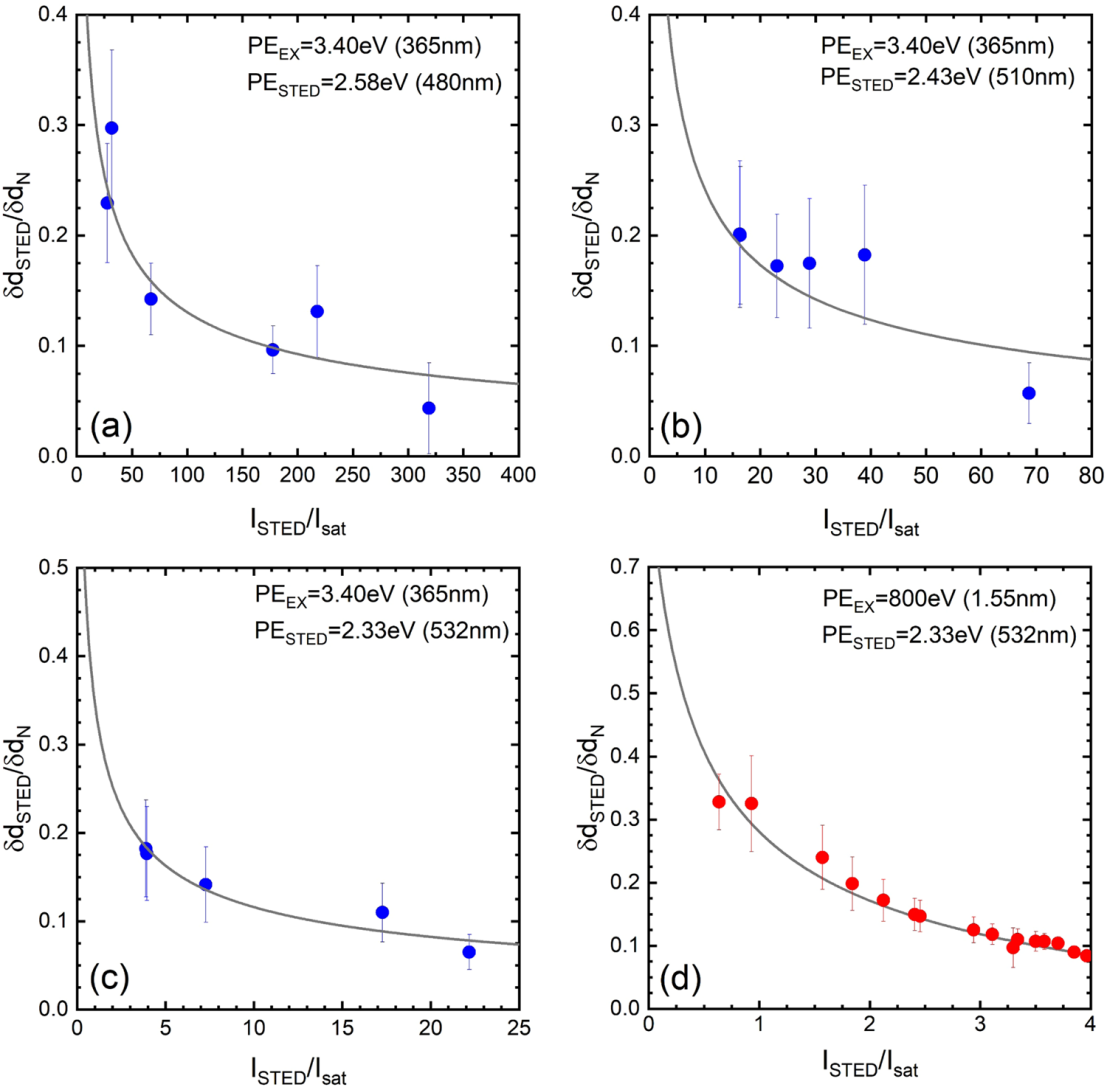
Normalized laser intensity dependences of the spot diameter ratio. The photon energies of the STED laser were (**a**) 2.58 eV (480 nm), (**b**) 2.43 eV (510 nm), and (**c**) 2.33 eV (532 nm), and the UV excitation light energy was 3.40 eV (365 nm). The photon energy of the STED laser was (**d**) 2.33 eV (532 nm), while that of the SX excitation light was 800 eV (1.55 nm). Circles with bars represent the measurement results with errors, and solid curves represent fitting results obtained using Eq. (1) as a guide for the eye.

Figure 4 shows the changes in the spot diameter ratio, which is the spot size of the luminescence at the dark centre of the STED light spot when normalized with respect to the STED laser spot diameter. In the figure, points with bars represent the measurement results and the curves represent the fitting results from Eq. (1). The spot diameter ratios decrease as the normalized laser intensity increases in all figures.

The spot diameter of the STED light in the beam profile shown in Fig. 3(b) was approximately 9.2 μm, while that of the luminescence light from the scintillator observed through the dark spot was 2.8 μm. Although the STED light diameter remained constant, even if the STED light intensity was changed, the diameter of the small bright spot increased as the luminescence intensity increased. The luminescence spot diameter also varied with the STED wavelength. The luminescence spot size changed from 1 to 4 μm when the STED photon energy was 2.58 eV, but changed from 2 to 8 μm at a photon energy of 2.25 eV. We observed a tendency where the normalized laser intensity decreased in tandem with reduction of the STED photon energy.

### Stimulated emission depletion by SX light excitation

The results in the previous section showed that the STED phenomenon occurred in the photon energy range from 2.58 eV (480 nm) to 2.25 eV (550 nm). We investigated the STED phenomenon in the SX-excited luminescence using the photon energy of 2.33 eV (532 nm) because of the ease with which suitable laser diodes (LDs) could be obtained. As a result, the same STED phenomenon and the same light intensity profile were obtained as in the UV excitation case, as shown in Fig. 3(c,d). Because the spot size of the incident SX light was smaller than that of the UV light, the black area at the bottom of Fig. 3(c) represents the weakly irradiated area. In the figure, light reflection from the scintillator surface (opposite the side that was under X-ray irradiation) is observed and the spots are clearly separated. Figure 3(d) shows the results for the intensity profiles when evaluated in the same manner as Fig. 3(b). The normalized laser intensity dependence of the spot diameter ratio was also evaluated in Fig. 4(d). As in the UV excitation case, the spot size ratio for SX excitation decreased as the normalized laser intensity increased.

### Beam diameter and luminescence spot diameter

The STED wavelength at which the smallest luminescence spot beam diameter was obtained in the experiment was 480 nm. The effective NA was 0.067 when taking the incident light beam diameter into consideration and the spot diameter was 4.4 μm at the Abbe diffraction limit predicted using this NA value. The actual STED light beam diameter was approximately equal to this expected value. The luminescence spot diameter was 1 μm, which was approximately 1/4 of the value determined from the Abbe diffraction conditions. By performing the same evaluation for the SX-excited STED, the luminescence spot diameter was shown to be approximately 1/10 of the spot diameter of the STED light.

## Discussion

The diameter of the luminescence spot within which the luminescence area is limited by the STED phenomenon is affected by two parameters: the size of the luminescence region along the depth direction from the surface upon which the X-rays are incident and the depth of focus (DOF) of the objective lens. The luminescence region along the depth direction is considered to be almost equal to the X-ray penetration depth, and the X-ray penetration depth is dependent on the photon energy of that X-ray. These penetration depths are less than 1 μm in most materials when the photon energy of the incident X-ray is less than 1 keV. In the case of Ce:LSO, however, the penetration depth remains less than 1 μm when the photon energy is less than 3 keV^7^. The DOF of the objective lens is expressed using Berek’s formula^8^. The objective lens used in this study has an NA of 0.2 and the observation wavelength of approximately 400 nm (Fig. 2); therefore, the value of the DOF is calculated to be 5.9 μm. This DOF is sufficiently longer than the attenuation length of Ce:LSO at the photon energy of 800 eV. In addition, the DOF of the objective lens used for observation of the Ce:LSO luminescence is 1 μm or less when the NA value is greater than 0.95, but most commercially available, non-immersion objective lenses have NA values of less than 0.95. In summary, as long as the STED luminescence of Ce:LSO is observed using a non-immersion lens in the photon energy region below 3 keV, the size of the STED luminescence point remains unaffected by the X-ray penetration depth. In the photon energy region above 3 keV, the thickness of the STED scintillator should be reduced to be below the penetration depth of the incident X-rays.

The simplest microscopes that can be realized by combining scintillator luminescence with use of the STED phenomenon are used in radiography. The spatial resolution of a two-dimensional detector with the same configuration used in this research was reported to be 200 nm^9^. By replacing this detector with a detector that uses a STED scintillator, the spatial resolution value can then be increased significantly. However, the light intensity detected by a pixel will decrease in inverse proportion to the square of the pixel size. Therefore, when it is assumed that the pixel size of the detector decreases to 1/10 of its previous size, approximately 100 times the previous measurement time or use of a strong X-ray light source such as an undulator will be required to acquire the same number of photons. An SX image in which the spatial resolution was evaluated as 3 μm at a photon energy of 91.8 eV would require a photon number of 4.8 × 10^10^ photons/mm^2^/s^7^. Therefore, an SX image with spatial resolution of 20 nm will require a photon number of 1.1 × 10^15^ photons/mm^2^/s or more, based on our previous results. In addition, the STED point must be scanned to obtain an X-ray image when using the STED scintillator. Because the scanning method takes the same measurement time for each of the considerable number of pixels, a multiple point scanning procedure will be required to reduce the measurement time. Use of a commercially available Nipkow disk with 1000 pinholes will allow the measurement time to be reduced to 1/1000 of the initial value^10^.

Because the Ce(1) and Ce(2) atoms at the Lu(1) and Lu(2) sites where the Lu atoms have been replaced by Ce atoms, respectively, are considered to have Oh symmetry with the crystal structure^11^, the electronic structure of the Ce^3+^ ions splits into ^2^*E*_2g_ and ^2^*T*_2g_ in accordance with the crystal field splitting mechanism^12^. The Ce 4 *f* orbitals also split into the ^2^*F*_7/2_ and ^2^*F*_5/2_ states in accordance with the spin-orbit splitting mechanism^13^. The luminescence of the Ce atoms is caused by a dipolar transition from the lowest Ce 5*d* orbital to the Ce 4 *f* orbitals^14^ and thus peaks A and B of the luminescence spectrum shown in Fig. 2(a,b) can be assigned to ^2^*F*_7/2_ and ^2^*F*_5/2_ of the Ce(1) site, respectively. Similarly, peaks C and D are assigned to ^2^*F*_7/2_ and ^2^*F*_5/2_ of the Ce(2) site, respectively. These deconvolved peaks represented by A – D have different widths among their peaks and different split widths between the Ce(1) and Ce(2) sites (Table 1). These differences may be caused by differences between the electronic structures of the Ce sites.

The photon energy required to cause stimulated emission has the same value as the luminescence peak; therefore, the photon energy that is equal to the value at each peak position in Fig. 2 is required to cause stimulated emission in Ce:LSO. Because these peaks overlap each other, as indicated in Fig. 2, the contributions of these transitions to the stimulated emission will depend on the STED laser’s photon energy. The stimulated emission of Ce:LSO can thus be classified using the peak luminescence positions while considering their respective peak widths. When Ce:LSO is irradiated by light with photon energy of 2.58 eV, the transitions contribute to the stimulated emission at peaks C and D, i.e., to an overlap of the transitions Ce 5*d* → ^2^*F*_7/2_ and Ce 5*d* → ^2^*F*_5/2_ at the Ce(2) site. Similarly, in the case where stimulated emission is produced by light with photon energy of less than 2.50 eV, the transition is that of peak D, i.e., Ce 5*d* → Ce 4*f   *^2^*F*_5/2_, at the Ce(2) site. As the photon energy decreases, the stimulated emission intensity also decreases according to the peak luminescence intensity. The reason why the STED phenomenon was hardly observed under 1.97 eV light irradiation is believed to be that no *4 f* level was contributing to the luminescence. In summary, the number of transition levels that contribute to the stimulated emission decreases as the photon energy of the irradiation decreases. This decreasing number of transition levels may thus be the origin of the decreasing intensity of the normalized laser beam.

### Summary

An investigation of whether the luminescence of a Ce:LSO crystal excited by UV light would be depleted or not when using photons from the energy region from 2.58 eV (480 nm) to 1.97 eV (630 nm) was performed. The normalized laser intensity at which the saturated luminescence intensity normalized the laser light intensity was shown to decrease as the photon energy decreased. In addition, the normalized spot diameter ratio that represents the spatial resolution of the STED microscope increased as the normalized laser light intensity decreased. The spot diameter ratio was obtained by normalizing the luminescence spot diameter of Ce:LSO using the spot diameter of the illumination light. This spot diameter ratio increases as the normalized laser light intensity decreases and this behaviour is explained well by the modified Abbe diffraction limit. The reduction in the normalized laser light intensity is considered to be dependent on the reduction in the density of states of the Ce*4f* electrons that contribute to the luminescence of Ce:LSO.

The STED phenomenon was also observed in the luminescence of Ce:LSO when excited by soft X-rays. In this case, the normalized laser light intensity at SX excitation was smaller than the UV excitation value, which caused the spot diameter ratio of the luminescence to increase. These results show that a scintillator using the STED phenomenon can be applied as a high-resolution two-dimensional detector in the X-ray region.

## Methods

To identify the Ce:LSO luminescence levels, the SX-excited luminescence spectrum was measured. It was then determined whether laser-induced emission depleted the Ce:LSO luminescence when the luminescence was excited using UV light. The STED phenomenon’s wavelength dependence was also investigated by varying the laser wavelength. Finally, the Ce:LSO luminescence excited by soft X-rays was confirmed to be suppressed, as in the UV excitation case.

### Luminescence spectra of Ce:LSO

The SX-excited luminescence spectrum was measured using optics evaluation beamline BL11D, Photon Factory, KEK^15^. The experimental setup and method were the same as in our previous paper^7^. Because the Photon Factory was operating in “Storage Mode,” where the ring current decreased with time, the incident SX intensity decreased with time because it was proportional to the ring current. Because the luminescence spectra were normalized using the incident SX light intensity for comparison, incident SX intensities were measured before and after energy distribution curve (EDC) measurement of the luminescence intensity; these intensities were averaged as the SX intensity of *I*_0_(*E*). Finally, the luminescence intensity EDC, *I*_*m*_(*E*), was normalized using exposure time *t*, *I*_0_(*E*), the reflectance and transmittance of the optical elements, and the spectrometer quantum efficiency. The luminescence spectrum *I*_*L*_(*E*) was obtained^7^.

### STED light source

A diode-pumped solid-state (DPSS) laser diode (532 nm, 40 mW; DJ 532–40, Thorlabs) was used as the STED light source for SX excitation measurement. A high-brightness supercontinuum (SC) generator was used as the STED light source for 3.40 eV excitation measurement^16^. The initial pulse was produced using a typical all-normal-dispersion (ANDi) mode-locked fibre oscillator^16,17^. Output pulses were detected using a Pockels cell in a regenerative amplifier (RGA). The Yb:YAG thin-disk RGA output power was 0.3 W at a 1 kHz repetition rate. The 1 kHz pulse was compressed using a transmission grating compressor and chirped mirrors to 300 fs at a 1030 nm carrier wavelength. To produce the supercontinuum beam, a 500 fs pulse was propagated through a 2-cm-long SiO_2_ crystal column. Typical supercontinuum beam spectra ranged from 400 to 1000 nm, and an interferometric filter was used at 532 nm.

The laser wavelength was confirmed using a spectrometer (Bluewave, Stellar Net) after passing through the vector optical system. The laser light intensity was adjusted using a power meter (PDA 200 C, Thorlabs) and measured using a microscope camera (DS-Fi1, Nikon) after passing through an optical filter.

### Optical system and vector beam converter

**Figure 5:**
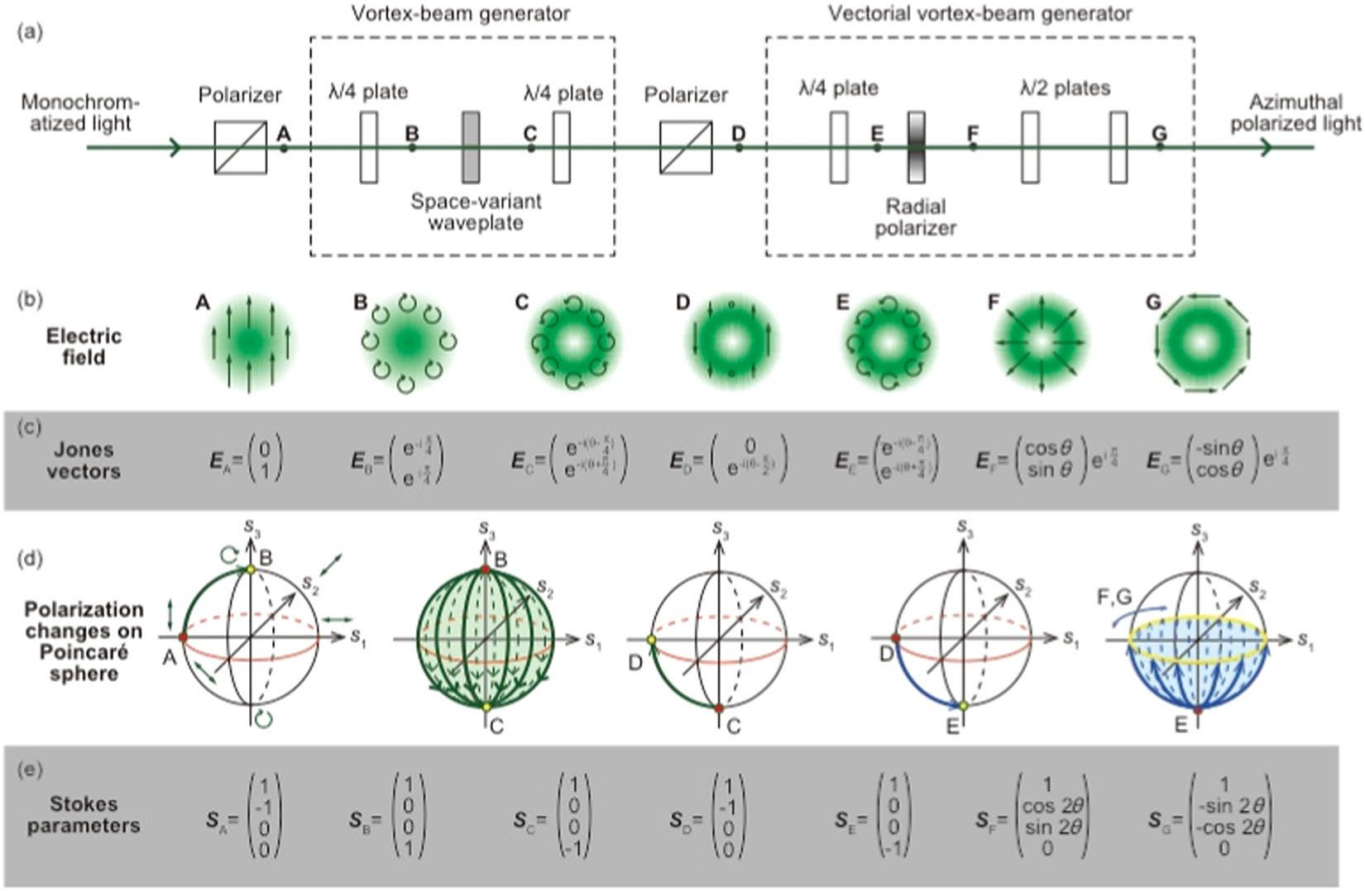
Concept of STED beam with azimuth polarization reducing the geometric phase. (**a**) Optical configuration of STED beam with azimuth polarization and uniform phase. (**b**) Electric field of the generated beams as determined using Jones calculus at points “A” to “G” in Fig. 5(a). (**c**) Jones vectors of generated beams. (**d**) Cyclic polarization changes caused by the polarization elements in Fig. 5(a) generate the geometric phase. A Poincare sphere is used for the geometric phase calculation.

Laser light was launched into the vector light system after being monochromatized using a wavelength filter. Figure 5 shows the STED beam concept, with azimuth polarization reducing the geometric phase. The optical configuration for the STED beam with azimuth polarization and uniform phase, consisting of a vortex beam generator and a vectorial vortex beam generator, is shown in Fig. 5(a). The vortex beam generator comprises two quarter-wave plates and a space-variant wave plate, which were fabricated as sub-wavelength structures on SiO_2_ plates. The vectorial vortex beam generator incorporates a quarter-wave plate, a radial polarizer and two half-wave plates as polarization rotators. Fig. 5(b) shows the electric fields of the generated beams, which were determined via Jones calculus at points “A” to “G” in Fig. 5(a). The beam at points “A” and “B” has a Gaussian profile. After passing through the space-variant wave plate, a doughnut-shaped beam is generated using the left-hand vortex phase of −*iθ* at point “C.” A left-hand circularly polarized beam is generated using the quarter-wave plate at point “E.” The vortex phase is cancelled by the inverse vortex phase generated by the radial polarizer. The vector beam is radially polarized. The polarization rotator, which consists of two half-wave plates, converts it into an azimuthally polarized beam with uniform π/4 phase. The Jones vectors of the generated beams are shown in Fig. 5(c). The phase factor of exp [−*i*(*θ* − π/4)] indicates another geometric phase caused by cyclical polarization element changes. After passing through the radial polarizer, the vector beam becomes radially polarized with uniform phase of exp (−*i*π/4). The phase factor of exp (−*iθ*) is cancelled by the inverse optical vortex. Finally, the polarization rotator is adjusted to be azimuthally polarized with uniform phase. Cyclic polarization changes caused by polarization elements shown in Fig. 5(a) generate a geometric phase, as shown in Fig. 5(d). A Poincare sphere is used for geometric phase calculations. Polarization changes from “A” to “G” are described on the Poincare sphere. After passing through “C,” the generated geometric phase is illustrated by the green area on the sphere. The geometric phase caused by the right-hand circularly polarized beam is 2π rad, because it is half of the sphere’s surface area. However, the geometric phase caused by the left-hand circularly polarized beam is also 2π rad. The geometric phase illustrated as the blue area is generated via two rounds on yellow circuits. The two vortex phases cancel each other.

### Sample preparation

The commercial Ce:LSO sample was optically polished to 0.2 mm thickness and had dimensions of 5 × 5 mm^2^.

### SX beamline

The SX light source for SX-excited STED experiments was beamline BL11D, as used in the luminescence spectral measurements^7^. Because the Photon Factory operated in “Top-Up Mode,” the ring current was constant and the incident SX intensity remained constant. Therefore, no normalization was performed using the incident SX intensity in the STED experiment. The incident SX light wavelength resolution was *λ*/*δ*λ = 1000. A four-dimensional slit was installed in the beamline and adjusted to produce a sufficiently small luminescence point.

### Microscope system

**Figure 6:**
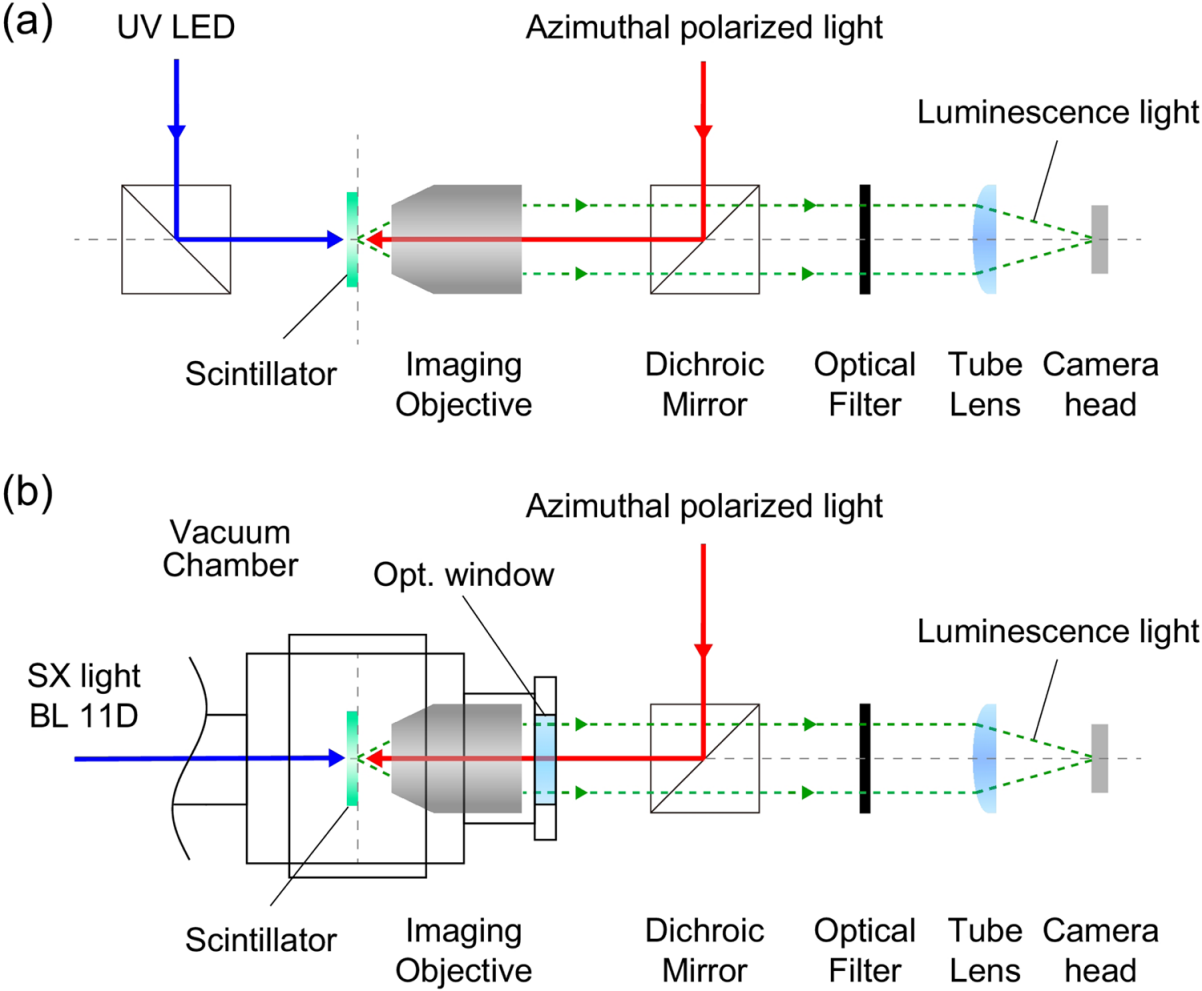
Layout of optics used in (**a**) the microscope unit for UV excitation and (**b**) the microscope unit for SX excitation.

For STED light spot reduction and scintillator image enlargement, a self-made microscope system was used, as shown in Fig. 6(a,b). The reflected STED light and the scintillator luminescence were separated from the sampled light using a dichroic mirror (DMSP 425, Thorlabs) and an optical filter (FGL 400, Thorlabs) inserted between an infinity-corrected objective (NA = 0.2; UV10X, Union Optical) and an imaging lens (UV Tube lens, Union Optical). The camera head (DS-Fi1, Nikon) for image detection has 2560 × 1920 pixels and deposited colour filters. The camera head colour filter operates in the blue (390–510 nm), green (480–600 nm), and red (580–680 nm) wavelength ranges when transmittance of 0.3 is the boundary condition. The camera head can separate the image colours and thus can separate the excitation light and the luminescence light if their colours are different. The intensities of the STED light and the scintillator luminescence were normalized using the measurement time and the number of pixels. The STED light beam diameter on entering the objective was 6 mm for 3.40 eV excitation and 4 mm for SX excitation. The effective NA of the objective was 0.067 under UV excitation and 0.044 under SX excitation.

### Measurements of stimulated emission depletion in Ce:LSO

**Figure 7:**
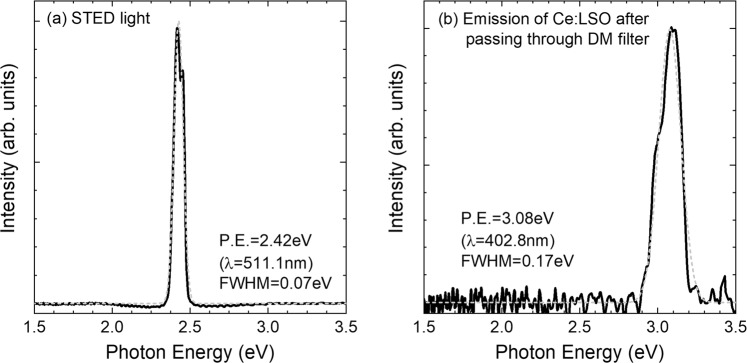
(**a**) Supercontinuum laser light after monochromatization. (**b**) Emission spectrum of Ce:LSO after passing through the DM filter.

This section describes the STED experiments on the scintillator using a supercontinuum laser and vector optics. The lasers and optics used are described elsewhere^18,19^. An interference filter monochromatized the supercontinuum laser light. When the laser photon energy was adjusted to 2.43 eV (*λ* = 510 nm), Fig. 7(a) shows the light intensity spectrum measured via a spectrometer (Bluewave, Stellar Net). The peak photon energy observed was 2.43 eV (*λ* = 511 nm) and the width was 0.07 eV (FWHM), as determined by Gaussian peak fitting. The estimated photon energy error was ±0.1 eV (±2 nm).

**Figure 8:**
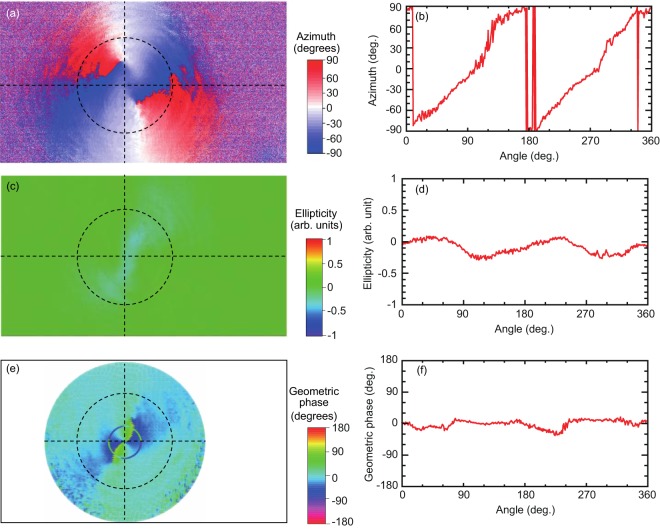
Evaluation of the generated azimuthally polarized beam via polarization analysis. (**a**,**c**) show the azimuth and ellipticity of the generated vector beam, respectively. The cross-sections at the broken lines in (**a**,**c**) are described in (**b**,**d**), respectively. For the azimuth and ellipticity, the geometric phase is determined as shown in (**e**). (**f**) Shows the cross-sectional profile of the obtained geometric phase. As a result, the generated vector beam is shown to have azimuthal polarization and uniform phase.

Supercontinuum laser light with a 400–1000 nm wavelength width was introduced into the vector-light generation optics and evaluation showed an elliptical distribution and the major-axis direction distribution of the exit light. Figure 8 shows evaluation of the azimuthally polarized beam performed via polarization analysis. The elliptical distribution of the measured light was ≤0.3 and the major-axis direction distribution showed that the light was azimuthally-polarized. The correlation percentage between measured and ideal values was ≥90%. Stokes parameters were measured by controlling the geometrical phase based on the presence or absence of light-vortex generation optics^18,19^. Evaluation of the geometrical phase from the Stokes parameters showed a uniform phase and a clockwise eddy phase.

The dichroic mirror described in section 2.2 can reflect light at wavelengths of 425 nm or more and guide it to the objective. The dichroic mirror and optical filter combination can transmit light with a 400–425 nm wavelength width and transmittance of ≥95% and transmits from the objective to the imaging lens. The light intensity of the 445–800 nm wavelength width is reduced to 1/50 or less by this combination. The scintillator light has two possible origins: the scintillator luminescence and reflection of the illuminating STED light from the scintillator. These two light beams have almost identical intensities after transmission using the dichroic mirror and optical filter combination and are separated into the STED light image and the luminescence image using the light filter on the camera head. The luminescence was measured via a spectrometer (Bluewave, Stellar Net) after the transmission, and Fig. 7(b) shows the spectrum. The luminescence light photon energy was 3.08 eV (*λ* = 402.8 nm) with a FWHM width of 0.17 eV (wavelength conversion value: 22.1 nm).

## Data Availability

The data generated and analysed during the current study will be made available from the corresponding author on reasonable request.
